# Supplementary material for: Assessing the validity of post-discharge readmission and mortality as a composite outcome among newborns in Uganda
Source: PLoS One. 2026 Feb 3;21(2):e0332787. doi: 10.1371/journal.pone.0332787 (PMC12867235; doi:10.1371/journal.pone.0332787)
Supplement: S1 Table — (DOCX) [file pone.0332787.s001.docx]

Assessing the validity of post-discharge readmission and mortality as a composite outcome among newborns in Uganda: Supporting Information

**S1 Table.** Subhazards ratios for post-discharge neonatal mortality or readmission from Fine-Gray models, and hazards ratios for the composite outcome from Cox proportional hazards models (in full).

| **Variable** | **Post-discharge mortality (95% CI)** | **Post-discharge readmission (95% CI)** | **Composite outcome (95% CI)** |
| --- | --- | --- | --- |
| ***Demographics and Social Variables*** |  |  |  |
| Age of Mother (per year) | 0.93(0.87 to 0.99) | 0.98(0.96 to 1.00) | 0.97 (0.95 to 1.00) |
| Age of the Mother: 18 – 35 years (ref. <18 years) | 0.78(0.10 to 5.77) | 0.62(0.32 to 1.19) | 0.63 (0.33 to 1.19) |
| Age of the Mother: Over 35 years (ref. <18 years) | 0.24(0.01 to 3.94) | 0.32(0.13 to 0.77) | 0.31 (0.13 to 0.72) |
| Travel time to hospital: 30 minutes – 1 hr (ref. <30 minutes) | 0.57(0.25 to 1.32) | 0.84(0.64 to 1.10) | 0.81 (0.63 to 1.04) |
| Travel time to hospital: > 1 hr (ref. <30 minutes) | 1.4(0.58 to 3.35) | 0.28(0.15 to 0.51) | 0.40 (0.24 to 0.66) |
| Number of people living in household | 0.82(0.67 to 1.00) | 1.00(0.95 to 1.06) | 0.98 (0.93 to 1.04) |
| Number of children living in household (including the new baby) | 0.82(0.64 to 1.04) | 0.99(0.92 to 1.07) | 0.97 (0.90 to 1.04) |
| Lives with the father of this baby | 3.09(0.42 to 22.68) | 1.13(0.71 to 1.81) | 1.23 (0.78 to 1.95) |
| Insufficient food | 0.31(0.07 to 1.30) | 1.65(1.24 to 2.20) | 1.45 (1.09 to 1.93) |
| Previous children who have died | 0.56(0.13 to 2.37) | 1.21(0.82 to 1.78) | 1.12 (0.77 to 1.64) |
| Socioeconomic Index Score (numeric) | 0.87(0.72 to 1.05) | 1.02(0.96 to 1.09) | 1.00 (0.94 to 1.07) |
| Socioeconomic Index Score: Moderate (ref. Low) | 0.89(0.42 to 1.87) | 1.28(0.96 to 1.71) | 1.22 (0.94 to 1.60) |
| Socioeconomic Index Score: High (ref. Low) | 0.57(0.20 to 1.57) | 1.04(0.73 to 1.47) | 0.97 (0.69 to 1.34) |
|  |  |  |  |
| ***Admission Characteristics*** |  |  |  |
| Not delayed > 1 hour | 2.29(0.54 to 9.57) | 1.73(1.08 to 2.77) | 1.79 (1.14 to 2.80) |
| Referral visit | 1.66(0.84 to 3.30) | 0.58(0.44 to 0.76) | 0.67 (0.52 to 0.86) |
| Mother here for elective c-section | 0.77(0.19 to 3.07) | 0.41(0.20 to 0.86) | 0.45 (0.23 to 0.87) |
| Time between labor and delivery (per hour) | 0.99(0.98 to 1.00) | 1(0.99 to 1.00) | 0.99 (0.99 to 1.00) |
| Vitals recorded | 22570.92(15165.71 to 33591.98) | 0.60(0.27 to 1.34) | 0.68 (0.30 to 1.55) |
| Systolic blood pressure (mmHg) | 0.99(0.96 to 1.01) | 1.00(0.99 to 1.01) | 1 (0.99 to 1.00) |
| Diastolic blood pressure (mmHg) | 1.01(0.98 to 1.03) | 0.99(0.98 to 1.01) | 1 (0.98 to 1.01) |
| Temperature at admission (°C) | 0.69(0.30 to 1.63) | 0.78(0.54 to 1.13) | 0.77 (0.56 to 1.06) |
| Heart rate (beats per minute) | 0.99(0.97 to 1.01) | 1.00(0.99 to 1.01) | 1.00 (0.99 to 1.01) |
|  |  |  |  |
| ***Pregnancy History*** |  |  |  |
| Prior C-section: <18 months ago (ref. no c-section) | 4.35(1.04 to 18.27) | 0.25(0.03 to 1.85) | 0.69 (0.22 to 2.18) |
| Prior C-section: 18-36 months ago (ref. no c-section) | 0.70(0.16 to 2.97) | 0.46(0.25 to 0.85) | 0.49 (0.28 to 0.85) |
| Prior C-section: >36 months ago (ref. no c-section) | 0.69(0.21 to 2.29) | 0.41(0.24 to 0.70) | 0.44 (0.27 to 0.72) |
| Prior history of poor milk production | 0.76(0.10 to 5.74) | 1.01(0.47 to 2.15) | 0.98 (0.48 to 2.00) |
| Number of ANC visits: 4 – 8 (ref. <4) | 1.25(0.54 to 2.88) | 1.23(0.90 to 1.67) | 1.23 (0.92 to 1.65) |
| Number of ANC visits: > 8 (ref. <4) | 0(0.00 to 0.00) | 1.10(0.40 to 3.06) | 0.97 (0.35 to 2.69) |
| High blood pressure (diagnosed before pregnancy) | 3.07(0.42 to 22.40) | 1.66(0.62 to 4.42) | 1.84 (0.75 to 4.48) |
| No prior diagnoses | 2.02(0.48 to 8.44) | 0.76(0.53 to 1.09) | 0.83 (0.58 to 1.18) |
| Due date known | 1.45(0.51 to 4.12) | 0.69(0.51 to 0.93) | 0.74 (0.55 to 0.99) |
| Gravidity | 0.77(0.60 to 0.98) | 1.00(0.93 to 1.07) | 0.97 (0.91 to 1.04) |
| Parity | 0.81(0.63 to 1.05) | 0.96(0.89 to 1.05) | 0.95 (0.87 to 1.02) |
| Pregnancy loss | 0.32(0.10 to 0.98) | 1.20(1.02 to 1.41) | 1.12 (0.95 to 1.33) |
| Diagnosed with preterm labour during this pregnancy | 2.70(0.37 to 19.69) | 3.04(1.52 to 6.07) | 3.02 (1.55 to 5.88) |
| Diagnosed with malaria during this pregnancy | 0.43(0.16 to 1.13) | 2.12(1.65 to 2.73) | 1.82 (1.43 to 2.32) |
| Diagnosed with urinary tract infection during this pregnancy | 0.22(0.07 to 0.63) | 1.82(1.41 to 2.34) | 1.49 (1.17 to 1.89) |
| Diagnosed with other infection during this pregnancy | 0.32(0.04 to 2.38) | 1.81(1.26 to 2.58) | 1.61 (1.13 to 2.29) |
| Not diagnosed with any infection during this pregnancy | 2.77(1.34 to 5.71) | 0.32(0.23 to 0.44) | 0.44 (0.34 to 0.59) |
| No known/suspected placental disorder | 0.36(0.11 to 1.20) | 2.20(0.81 to 5.92) | 1.41 (0.66 to 2.99) |
| No sure known/suspected placental disorder (this pregnancy) | 1.96(0.46 to 8.19) | 0.25(0.06 to 1.01) | 0.44 (0.16 to 1.19) |
| Medical history of high blood pressure | 1.16(0.15 to 8.47) | 1.8(0.98 to 3.28) | 1.72 (0.96 to 3.08) |
| Medical history of HIV | 2.03(0.71 to 5.77) | 1.11(0.68 to 1.81) | 1.22 (0.78 to 1.91) |
| Medical officer provided ANC care | 0.54(0.07 to 3.96) | 1.32(0.81 to 2.16) | 1.22 (0.76 to 1.98) |
| Traditional birth attendant provided ANC care | 1.54(0.21 to 11.34) | 3.13(1.84 to 5.33) | 2.97 (1.76 to 5.01) |
|  |  |  |  |
| ***Delivery (Maternal)*** |  |  |  |
| Number of babies delivered | 2.12(0.67 to 6.69) | 0.45(0.18 to 1.09) | 0.64 (0.31 to 1.30) |
| Caesarean delivery | 1.24(0.61 to 2.50) | 0.30(0.21 to 0.44) | 0.38 (0.28 to 0.53) |
| Woman was given an episiotomy | 1.20(0.46 to 3.12) | 1.04(0.71 to 1.50) | 1.06 (0.74 to 1.50) |
| Postpartum haemorrhage | 3.31(1.01 to 10.85) | 0.83(0.37 to 1.86) | 1.11 (0.57 to 2.17) |
| No. of blood units transfused (ref. No transfusion) | 1.66(1.32 to 2.09) | 1.08(0.78 to 1.50) | 1.28 (1.04 to 1.58) |
| Labour obstructed | 2.42(1.09 to 5.36) | 0.32(0.17 to 0.60) | 0.52 (0.32 to 0.84) |
| Meconium in the amniotic fluid during labour | 1.65(0.26 to 10.52) | 0.29(0.07 to 1.19) | 0.45 (0.15 to 1.37) |
| Manual removal of placenta | 1.35(0.32 to 5.62) | 0.80(0.41 to 1.56) | 0.86 (0.47 to 1.59) |
| Vaginal or perineal tearing: Degree 1 (ref. None) | 1.14(0.34 to 3.74) | 0.88(0.52 to 1.49) | 0.91 (0.56 to 1.49) |
| Vaginal or perineal tearing: Degree >=2 (ref. None) | 0.38(0.05 to 2.80) | 2.43(1.73 to 3.41) | 2.16 (1.54 to 3.02) |
| Number of vaginal exams: 3-5 (ref. 0-2) | 0.46(0.19 to 1.07) | 0.77(0.58 to 1.02) | 0.72 (0.55 to 0.95) |
| Number of vaginal exams: >5 (ref. 0-2) | 0.88(0.30 to 2.56) | 0.98(0.65 to 1.48) | 0.97 (0.66 to 1.42) |
| Surgical Urgency: Delayed C section (ref. Vaginal Birth) | 1.34(0.53 to 3.33) | 0.30(0.17 to 0.52) | 0.39 (0.24 to 0.62) |
| Surgical Urgency: Timely C section (ref. Vaginal Birth) | 1.17(0.49 to 2.78) | 0.31(0.19 to 0.49) | 0.38 (0.25 to 0.57) |
| Gestation period: Under 37 weeks | 1.37(0.25 to 7.49) | 1.17(0.72 to 1.91) | 1.19 (0.74 to 1.90) |
| Gestation period: 37 - 40weeks | 1.47(0.48 to 4.48) | 0.67(0.48 to 0.95) | 0.73 (0.52 to 1.01) |
| Gestation period: Over 40 weeks | 1.43(0.46 to 4.40) | 0.62(0.44 to 0.88) | 0.68 (0.48 to 0.94) |
|  |  |  |  |
| ***Delivery (Neonatal)*** |  |  |  |
| Female sex | 1.07(0.53 to 2.13) | 0.96(0.74 to 1.24) | 0.97 (0.76 to 1.23) |
| Apgar score after 1 minute | 0.82(0.65 to 1.04) | 0.98(0.86 to 1.12) | 0.95 (0.84 to 1.07) |
| Apgar score after 5 minutes | 0.81(0.63 to 1.04) | 1.09(0.79 to 1.50) | 1.02 (0.81 to 1.30) |
| Birth weight (kg) | 0.33(0.09 to 1.11) | 0.87(0.66 to 1.15) | 0.78 (0.53 to 1.14) |
| Birth weight <2.5kg | 7.25(3.63 to 14.48) | 1.46(1.02 to 2.08) | 0.51 (0.38 to 0.70) |
| Length (cm) | 0.84(0.67 to 1.07) | 0.91(0.83 to 0.99) | 0.90 (0.83 to 0.97) |
| Resuscitation at birth | 0.13(0.01 to 1.19) | 2.71(2.09 to 3.50) | 2.16 (1.69 to 2.77) |
|  |  |  |  |
| ***Maternal Discharge*** |  |  |  |
| Mother admitted to higher level of care after delivery | 1.33(0.31 to 5.58) | 1.29(0.77 to 2.19) | 0.76 (0.46 to 1.25) |
| Systolic blood pressure | 0.97(0.95 to 1.00) | 1.00(0.99 to 1.01) | 1.00 (0.99 to 1.01) |
| Diastolic blood pressure | 0.99(0.96 to 1.02) | 1.00(0.99 to 1.01) | 1.00 (0.99 to 1.01) |
| Respiratory rate (breaths per minute) | 0.90(0.82 to 0.98) | 1.04(1.01 to 1.08) | 1.03 (1.00 to 1.06) |
| Temporal artery temperature (°C) | 1.34(0.77 to 2.33) | 1.04(0.74 to 1.48) | 1.08 (0.78 to 1.48) |
| Best SpO^2^ of mother | 1.73(1.26 to 2.36) | 0.85(0.80 to 0.91) | 0.87 (0.82 to 0.93) |
| Best heart rate (beats per minute) | 1.01(0.99 to 1.03) | 1.00(0.99 to 1.01) | 1.00 (0.99 to 1.01) |
| Hematocrit (%) | 0.96(0.89 to 1.04) | 1.00(0.98 to 1.03) | 1.00 (0.97 to 1.02) |
| Mother given oral antibiotics | 3.35(1.38 to 8.11) | 0.33(0.25 to 0.44) | 0.43 (0.34 to 0.56) |
| Mother given IV antibiotics | 1.38(0.69 to 2.78) | 0.33(0.23 to 0.48) | 0.42 (0.30 to 0.58) |
| No antibiotics | 0.33(0.12 to 0.86) | 3.14(2.42 to 4.07) | 2.48 (1.95 to 3.16) |
|  |  |  |  |
| **Neonatal Discharge** |  |  |  |
| Baby pooped | 1.16(0.35 to 3.80) | 0.73(0.50 to 1.05) | 0.76 (0.53 to 1.09) |
| Baby peed | 2.17(0.29 to 16.18) | 1.59(0.80 to 3.14) | 1.64 (0.86 to 3.13) |
| Mean temperature (°C) | 0.71(0.39 to 1.28) | 1.37(1.14 to 1.63) | 1.29 (1.06 to 1.56) |
| Respiratory rate (breaths per minute) | 0.96(0.92 to 0.99) | 1.00(0.98 to 1.02) | 0.99 (0.98 to 1.01) |
| Mean best foot/rhand SpO^2^ of newborn | 1.03(0.94 to 1.14) | 0.91(0.89 to 0.94) | 0.92 (0.89 to 0.95) |
| Mean foot/rhand heart rate of newborn (beats per minute) | 1(0.97 to 1.02) | 1.01(1.00 to 1.02) | 1.01 (1.00 to 1.02) |
