# Supplementary material for: Assessing the validity of post-discharge readmission and mortality as a composite outcome among newborns in Uganda
Source: PLoS One. 2026 Feb 3;21(2):e0332787. doi: 10.1371/journal.pone.0332787 (PMC12867235; doi:10.1371/journal.pone.0332787)
Supplement: S2 File — (DOCX) [file pone.0332787.s002.docx]

Inclusivity in global research

PLOS’ policy on inclusivity in global research aims to improve transparency in the reporting of research performed outside of researchers’ own country or community and ensures that PLOS publications reporting global research adhere to high standards for research ethics and authorship. Authors of relevant research articles may be asked to complete the questionnaire below, which outlines ethical, cultural, and scientific considerations specific to inclusivity in global research. This questionnaire may be requested when researchers have travelled to a different country to conduct research, if research uses samples collected in another country, research with Indigenous populations or their lands, or if research is on cultural artefacts. Researchers travelling to another country solely to use laboratory equipment will not normally be required to complete the questionnaire. However, the questionnaire can be requested at the journal’s discretion for any submission – if you have been requested to complete this questionnaire by the PLOS journal you submitted to, please do so.

Please complete the questionnaire below and include this as a Supporting Information file with your manuscript. Note that if your paper is accepted for publication, this checklist will be published with your article in the supporting information files. Please ensure that you reference the checklist in the main body of your manuscript. We suggest adding a subsection ‘Inclusivity in global research’ to your Methods section and adding the following sentence: “Additional information regarding the ethical, cultural, and scientific considerations specific to inclusivity in global research is included in the Supporting Information (SX Checklist)”

The questions have been designed to be applicable to a wide range of study types, and there are subsections for both human subjects research and non-human subjects research. If any of the questions are not relevant to your research please mark them as “N/A” as appropriate.

**Ethical considerations, permits and authorship**

*This section is applicable to all research types.*

Provide details as to who granted permissions and/or consent for the study to take place in the Methods section of your manuscript. This should include the names of **all** ethics boards, governmental organizations, community leaders or other bodies that provided approval for the study. If individuals provided approval refer to these people by their role or title but do not list their name(s).

Reported on page number: 4

If there were any deviations from the study protocol after approval was obtained please provide details of these changes in the Methods section of your manuscript.
Did this study involve local collaborators that are residents of the country where the research was conducted or members of the community studied? If you do not have any authors from said communities, please provide an explanation for this below.

Reported on page number: NA

Yes, the study included authors Angella Namala, Joseph Ngonzi, Nathan Kenya Mugisha who are residents of the country where the research was conducted.

Everyone listed as an author should meet PLOS’ criteria for authorship and all individuals who meet these criteria should be included in the author byline, rather than the acknowledgements. For further information please see the journal’s Authorship Policy.

**Human subjects research (e.g. health research, medical research, cross-cultural psychology)**

Did you obtain written informed consent from a representative of the local community or region before the research took place? How did you establish who speaks for the community? Details of written informed consent obtained from study participants should be reported separately in the Methods section of your manuscript.

Informed consent was conducted as per the protocol approved by the IRBs both in Uganda and Canada. Community consent was not specifically obtained nor was it required. These regional referral hospitals serve approximately 20 districts and comprise over 1 million individuals from thousands of communities. Prior to study initiation it was not possible to know which communities would be represented by the enrolled mothers, since every community in the catchment was not represented in the facility deliveries that were enrolled into this study. While we appreciate the desire for community consent, we do not believe that such practices are feasible for studies such as this.

How did members of the local community provide input on the aims of the research investigation, its methodology, and its anticipated outcome(s)?

This study was led by researchers from Uganda and Canada. Our research team has been continuously present in the districts where the study took place for the past 15 years and indeed this study question was borne out of our discussions with leaders from the Ministry of Health and local obstetricians who themselves interact daily with members of the communities we have engaged with. Over the past 8 years we have held several stakeholder meetings which included community leaders and village health team members who have been engaged in our Smart Discharges program. This specific analysis focuses on a very narrow goal of understanding the dual nature of readmissions both as an undesirable outcome (recurrent illness) as well as a desirable outcome (appropriate health seeking). It is precisely because of our deep community engagement that we have been able to recognize that an outcome like newborn readmissions in Uganda is in no way similar to readmissions in Canada. Indeed, in the US or Canada we would desire to

see newborn readmission decrease while in Uganda we would like to see the opposite, ensuring that all cases of recurrent newborn illness receive care which is currently not the case. Our analysis is imperative in dissecting this important issue so that new initiatives to improve newborn health do not attach a western interpretation of surrogate outcomes such as readmissions (a simple pubmed search will quickly identify dozens of studies which use readmission in a negative light in settings similar to Uganda). Our work, built with robust engagement from a broad group of stakeholders (including community members) formed over the past 15 years affirms the relevance of our work within the communities where we work.

When engaging with the local community, how did you ensure that the informed consent documents and other materials could be understood by local stakeholders?

We worked with professional translators who ensured us, who have as a part of their contract, that these materials were presented in an understandable way. The REB in Uganda confirmed that these documents were appropriate for the context when they granted us approval. All research assistants were careful in presenting these materials to study participants.

Will the findings of the research be made available in an understandable format to stakeholders in the community where the study was conducted (e.g. via a presentation, summary report, copies of publications, etc.)? Please provide details of how this will be achieved.

The parent study (still under review, though currently available as a pre-print) will be made available in a variety of formats for stakeholders according to our initial dissemination plan (not part of this analysis, so will not get into details here). This specific secondary analysis was unfunded and has minimal resources for community-level dissemination, though summaries will be created and posted to our website. More importantly, these analyses will inform future interventional studies to ensure optimal outcome are used to measure the effect of an intervention. As previously mentioned, readmission is often used as an outcome to be avoided, though we have shown this not to be the case. As we all work towards improving newborn mortality, we must recognize that the very important tasks of community engagement, broad stakeholder representation and similar considerations all must serve a more important goal of improving health for the most vulnerable, in this case newborns in Uganda. For this specific analysis, therefore, a key stakeholder group are researchers who design interventional studies, policy makers and administrators who measure health outcomes, especially in maternal and child health. We will thus be presenting these findings (and similar findings from under-5 who were discharged following sepsis) at local, national and international conferences in Africa.

**Non-human subjects research using specimens/ animals collected as part of the study, or those housed in archival collections. Examples include archaeology, paleontology, botany and zoology.**

Did the permission you obtained from a local authority to perform the study include an agreement on access to outputs and benefit sharing? This may include procedures to enable fair distribution of the benefits and resources arising from the research performed. Please include any details of Prior Informed Consent and Benefit Sharing Agreements obtained. These may be required by field-specific regulations, for example the Convention on Biological Diversity (CBD) and the associated Nagoya Protocol.

NA

If the material used in your study was imported, please A) provide the year it was imported and B) indicate whether permits were obtained to import/export the materials used, C) provide details of any permits obtained. If this information is not available, please indicate this.

NA

If you used archival specimens, please state how the material used in your study was acquired by the institute it is held in and provide details of any permits obtained for the original excavations/ sample collection. If this information is not available, please indicate this.

NA

How was the potential cultural significance of the materials collected in your study to local communities considered in your research design? Were Indigenous peoples and/or local researchers and institutions involved with archaeological excavations / collection of specimens? If so, please provide a description of their involvement.

NA

If your manuscript includes photographs of human remains please indicate whether authors obtained permission from descendants or affiliated cultural communities to do so.

NA
